# Evaluation of a Population-Based Targeted Screening Approach for Skin Cancer with Long-Time Follow-Up in Austria including Potential Effects on Melanoma Mortality

**DOI:** 10.3390/cancers16071283

**Published:** 2024-03-26

**Authors:** Wolfgang Brozek, Patrick Clemens, Hanno Ulmer, Nina Häring, Hans Concin, Emanuel Zitt, Gabriele Nagel

**Affiliations:** 1 Agency for Preventive and Social Medicine, 6900 Bregenz, Austria; patrick.clemens@lkhf.at (P.C.); hanno.ulmer@i-med.ac.at (H.U.); hans.concin@aks.or.at (H.C.); emanuel.zitt@lkhf.at (E.Z.); gabriele.nagel@uni-ulm.de (G.N.); 2 Department of Radio-Oncology, Feldkirch Academic Teaching Hospital, 6800 Feldkirch, Austria; 3 Department of Medical Statistics, Informatics and Health Economics, Innsbruck Medical University, 6020 Innsbruck, Austria; 4 Department of Dermatology and Venerology, Feldkirch Academic Teaching Hospital, 6800 Feldkirch, Austria; nina.haering@lkhf.at; 5 Vorarlberg Institute for Vascular Investigation and Treatment (VIVIT), 6800 Feldkirch, Austria; 6 Department of Internal Medicine 3 (Nephrology, Dialysis and Hypertension), Feldkirch Academic Teaching Hospital, 6800 Feldkirch, Austria; 7 Institute of Epidemiology and Medical Biometry, Ulm University, 89081 Ulm, Germany

**Keywords:** skin cancer, melanoma, targeted screening, screening, epidemiology, prevention, mortality, Vorarlberg Health Monitoring and Promotion Program, VHM&PP

## Abstract

**Simple Summary:**

To date, it remains unclear whether population-based skin cancer screening lowers melanoma-specific mortality. We herein evaluated a population-based skin cancer program that followed a pragmatic targeted screening approach conducted in the Austrian province Vorarlberg in 1989–1994 and examined possible effects on melanoma mortality in the general population during follow-up until 2019. Relative to the general population and participants of a health examination program, invasive and in situ melanoma incidences, as well as melanoma mortality, were increased. In the general population of Vorarlberg, however, melanoma mortality declined until 2004, though statistically non-significantly. Arguments for and against a contribution of the program are considered. Given the uncertain effectiveness of expensive population-wide mass screening programs, targeted risk-based skin cancer screening could be considered a viable cost-effective alternative strategy to prevent melanoma deaths.

**Abstract:**

Background: whether screening for skin cancer affects melanoma-specific mortality in a population-based setting remains unclear. Methods: in this population-based cohort study, we characterized and evaluated a skin cancer prevention program following a targeted screening approach conducted in 1989–1994 in the Austrian province Vorarlberg, with follow-up until 2019. The general population and attendees of a health examination program served for comparison. Results: in the screening program including full follow-up until 2019, 207 invasive and 187 in situ melanomas were identified in 8997 individuals. Incidences of invasive and in situ melanomas were elevated compared to the general population (IRR 2.92, 95%-CI 2.49–3.41, and IRR 4.13, 95%-CI 3.53–4.83, respectively) and the health examination program (HR 3.02, 95%-CI 2.59–3.52, and HR 3.90, 95%-CI 3.30–4.61, respectively). Breslow thickness and Clark’s level at time of invasive diagnosis were significantly lower in 1989–2019, but the tumor characteristics of the melanomas diagnosed during 1989–1994 did not differ from the comparison groups. Moreover, melanoma mortality was significantly elevated in the screening program (IRR 1.66, 95%-CI 1.00–2.75 vs. the general population, HR 2.12, 95%-CI 1.25–3.61 vs. the health examination cohort). Melanoma mortality in Vorarlberg declined until 2004, though statistically non-significantly. Conclusions: given the uncertain effectiveness and high public expenditures of population-wide mass screening programs, primary prevention and targeted risk-based skin cancer screening might be promising alternatives.

## 1. Introduction

Global incidence of skin cancers such as malignant melanoma has risen considerably during the last decades [1,2,3]. In Austria, incidence (age-standardized according to the European standard population) increased by approximately one third from 15 to 20/100,000 during 2000–2017 [4]. Generally, the rise in melanoma incidence throughout the last decades has been ascribed to several factors, including changing leisure time activity patterns involving more sun exposure, as well as enhanced screening for skin cancer, altered criteria for histologic diagnosis, and more modern, optimized diagnostic options [1,3,5]. In addition, the progression of climate change could have contributed to the higher melanoma risk [6]. Even though a decelerated increase or even decrease in incidence rates is anticipated for the present decade in some countries including Denmark, New Zealand, and the US [3], the number of diagnoses is nonetheless expected to continue to rise in an aging population [7]. In contrast, age-standardized mortality rates for melanoma are reported to have slightly decreased between 1990 and 2019, globally [2], while another study including 31 countries found upward trends for male and varying tendencies for female mortality between 1985 and 2015 in most countries [8]. In Austria, mortality rates have been constant during the last two to three decades, ranging from 4.0 to 4.5/100,000 (age-standardized according to the European standard population) in most years [4].

Apart from periodical exposure to UV radiation (primarily UV-B) resulting in sunburns [9], further risk factors for the carcinogenesis of melanoma encompass a light skin type [10], family history of melanoma [10], number and size of moles [11,12], as well as advanced age and male sex [1]. Prognostic factors associated with worse survival rates from melanoma are advanced age, being male, and greater tumor thickness at melanoma diagnosis [13].

The goal of skin cancer prevention activities such as screening programs is risk factor identification to lower the risk of acquiring malignant skin disease and dying of it. Furthermore, screening should raise detection rates of tumors at an early stage, resulting in a better outcome. Indeed, tumor thickness at diagnosis has been reported to be lower in patients who had undergone a skin cancer screening activity [14]. Nonetheless, the usefulness of screening programs to curb melanoma mortality is subject to controversy. In this regard, while screening activities in a non-population-based setting showed a clear benefit [15], the introduction of a nationwide skin cancer screening program in Germany in 2008 has not resulted in a discernible effect on the population level thus far [16,17,18,19]. In view of conflicting results and insufficient evidence for a beneficial impact, independent expert groups currently do not either recommend or discourage skin cancer screening for melanoma prevention in the general population, such as the U.S. Preventive Services Task Force [20,21], or advocate in favor of screening only for risk populations, such as the Cancer Council Australia [22].

During 1989–1994, a skin cancer screening program involving a whole-body examination was offered to the adult population of Vorarlberg, the westernmost province of Austria. This activity was part of the Vorarlberg Health Monitoring and Promotion Program (VHM&PP), a large population-based medical prevention program consisting of a general health examination as its core constituent [23,24]. Since the skin cancer examination was also offered to participants of the health examination, a pre-selection of patients likely occurred, resulting in a population-based, risk-adjusted, targeted skin cancer screening cohort. The goals of the present study are (1) to analyze the development of epidemiologic and clinical parameters (melanoma incidence and mortality, tumor stage, and tumor thickness at diagnosis) in participants of the screening program vs. participants of the health examination without a skin screening examination and vs. the general population, (2) to examine a possible impact of the screening activity on melanoma mortality of the population, and (3) to evaluate the targeted screening approach, overall and by sex. Extensive follow-up until 2019 and availability of individual level data led us to expect potentially insightful results regarding the efficacy of population-based skin cancer screening programs.

## 2. Patients and Methods

### 2.1. The Skin Cancer Screening Program as Part of the Vorarlberg Health Monitoring and Promotion Program (VHM&PP)

The Vorarlberg Health Monitoring and Promotion Program (VHM&PP) is a voluntary population-based secondary prevention program in Vorarlberg, the westernmost Austrian province, for the prevention of foremost cardiovascular diseases and cancer [23,24]. As part of its core activities, 185,459 inhabitants, more than half of the residents of Vorarlberg during that period, were recruited to a free general health examination conducted by general practitioners between 1 January 1985 and 30 June 2005. The examination comprised a blood draw with quantification of blood serum parameters, blood pressure measurement, measurement of body weight and height, and documentation of smoking, marital, and occupational status. Electronic records of all health examinations were documented by the Agency of Preventive and Social Medicine (aks gesundheit). For the skin cancer screening cohort (henceforth “VHM-Skin”), a comparison cohort was constructed from participants in the health examination devoid of skin cancer screening (henceforth “VHM-Health Exam”).

In the framework of the VHM&PP, a skin cancer screening program was offered free of charge between 1 August 1989 and 31 December 1994 to all inhabitants of Vorarlberg over 20 years of age, carried out by all registered dermatologists in the province during that time (n = 6) [25]. Though open to all adults, the program was offered explicitly to attendees of the health examination where general practitioners checked the participants’ skin status according to eczema, ulcers, and suspected pre-cancerous and neoplastic lesions. Patients with suspected lesions were referred to the participating dermatologists. Given the high participation rate in the health examination [23,24], pre-selection of individuals at high risk for skin cancer can be assumed, corresponding to a real-world targeted screening approach. Participants of the skin cancer program underwent a whole-body examination using dermoscopy, also including examination of the oral mucosa and superficial lymph nodes, and advice was given on sun-exposure-related risk of melanoma. In general, follow-up examinations were recommended every other year; however, participants identified as vulnerable risk patients could be summoned semi-annually or annually. In order to determine whether a patient was at a high or low risk of developing malignant skin cancer, a predefined questionnaire was used to assess the patient’s personal and family history of melanoma and NMSC (non-melanoma skin cancer), as well as any risk factors (i.e., UV exposure as reported by the participant, number of normal and dysplastic nevi, prevalent skin lesions, and skin damage caused by X-ray radiation). Today, this approach continues to be employed [26]. Follow-up visits for a lesion detected at a preceding screening examination or elsewhere were treated as routine examinations and not billed as part of the screening program. All data acquired on clinical diagnosis, skin type, and skin cancer risk (high risk yes/no, data on individual risk variables were not available) were documented and recorded electronically by the Agency of Preventive and Social Medicine (aks gesundheit). Due to lack of further funding, the skin cancer program was discontinued after 1994, and skin examinations were offered to the general population as an individual health service for a fee. Until 2023, no other publicly paid screening programs were conducted in Vorarlberg. In total, 9382 individuals participated in 12,069 examinations as part of the skin cancer screening program.

### 2.2. Study Design

**Figure 1:**
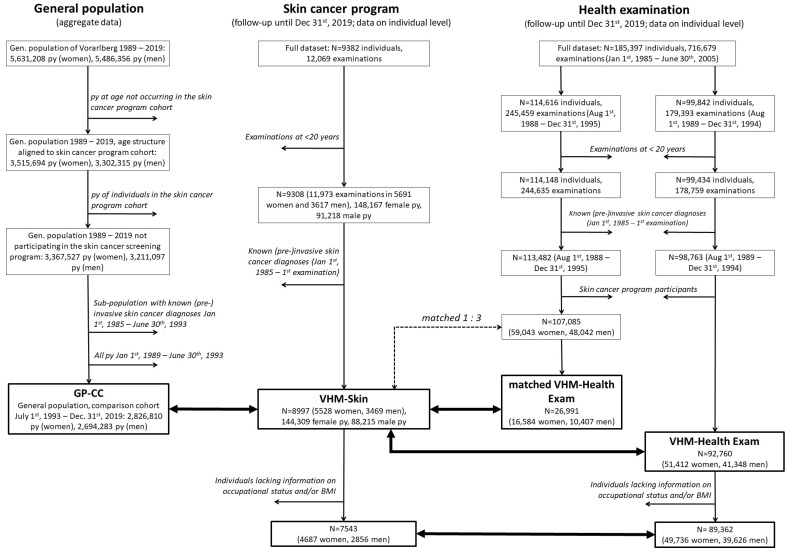
Flow chart of the study design. Thick double arrows connect cohorts framed in bold that were subjected to comparative analyses; py, person-years.

A flow diagram detailing the design of this population-based cohort study is shown in Figure 1. The minimum age for study entry was 20 years, and (unintentional) examinations below that age were excluded from the skin cancer program and health examination datasets. Entry in the study was the date of the first examination as of 1 August 1989. Individuals with known prevalent (pre-)invasive skin cancer diagnosed between 1 January 1985 and the date of study entry were excluded from all study groups. These diagnoses, obtained from the Cancer Registry Vorarlberg, included International Classification of Disease (ICD)-9 codes 172, 173, 232, and 238.2 corresponding to converted ICD-10 codes C43 (malignant melanoma of the skin), D03 (melanoma in situ), C44 (other neoplasms of the skin), D04 (carcinoma in situ of the skin), and D48.5 (neoplasms of the skin with uncertain or unknown behavior). The final VHM-Skin cohort (n = 8997, 232,524 person-years until 2019) was compared with the health examination cohort as well as the general average annual population of Vorarlberg between 1989 and 2019, excluding participants in the skin cancer program.

Comparability with aggregate data of the general population was achieved by subtracting each year’s person-years at all ages of the skin cancer program participants from the average annual general population. In addition, diagnoses and melanoma deaths occurring in the VHM-Skin cohort were subtracted based on cancer registry data. Date of study entry was uniformly set to 1 July 1993, as the mean time to study entry in VHM-Skin was 8.5 years from 1 January 1985. Person-years of prevalent cases identified between 1 January 1985 and 30 June 1993 were subtracted from all following years until the end of the study (31 December 2019) or until the date of death. All person-years before 1 July 1993 were excluded. Likewise, person-years at ages absent from the VHM-Skin in each year were removed to ensure compatibility of age structures, resulting in constantly rising age after 1994 in the final comparison cohort from the general population (henceforth “GP-CC”, 5,521,093 person-years until 2019).

The full health examination data set was edited in two ways in order to construct cohorts for comparison with the VHM-Skin cohort. (1) The full data set was restricted to the participants’ 1st examination between 1 August 1989 and 31 December 1994 at ≥20 years of age, including only individuals lacking known prevalent cases and who did not attend the skin cancer program (n = 92,760), yielding VHM-Health Exam. (2) As a sensitivity analysis, a matched sample was set up: the full data set was restricted to the participants´ 1st examination between 1 August 1988 and 31 December 1995 (because examination dates of matched cases were allowed to diverge by 1 year) at ≥20 years of age that defined their study entry, including only individuals lacking known prevalent cases and who did not attend the skin cancer screening program (n = 107,085). Matching to VHM-Skin (n = 8997) was by 3:1, according to sex, age at examination (±0.1 years), and examination date (±1 year), yielding a final cohort size of n = 26,991 (matched VHM-Health Exam).

### 2.3. Exposure and Covariates

The exposure variable was participation vs. no participation in the skin cancer program between 1 August 1989 and 31 December 1994. Moreover, the time period in 5-year intervals between 1985 and 2019 was used as the exposure variable in regression models evaluating temporal changes in incidence and mortality in the general population. Covariates in aggregate data of the general population comprise sex, age group (20–29, 30–39, 40–49, 50–59, 60–69, 70–79, 80+ years when compared with the VHP-Skin Cohort, and additionally 0–19 years in the entire general population), and time period (1989–1994, 1995–1999, 2000–2004, 2005–2009, 2010–2014, 2015–2019). Analyses at the individual level were adjusted for sex and age at study entry. For additional sensitivity analysis, further covariates mentioned in Section 2.1*.* were available for those who also took part in the health examination, with occupational status (as a proxy for socio-economic status) included for its reported role in participation rates and delay in melanoma diagnosis [27,28] and BMI (as continuous variable) included as a potential risk factor for melanoma carcinogenesis, at least in men [29]. In case of participation in more than one health examination, data from the one examination with the least temporal deviation from the first skin examination were selected. Categories of occupational status are blue collar worker, white collar worker, and self-employed; persons retired at study entry were categorized according to their former profession, while jointly insured family members were categorized according to the insured person. Owing to additional participation in the health examination, 7543/8997 (84%) members of the VHM-Skin cohort had complete records of both covariables, and complete records were ascertained in 89,362/92,760 (96%) members of the VHM-Health Exam cohort (Table S1). We abstained from further matching of the VHM-Skin sub-cohort with complete records on occupational status and BMI (n = 7543) with the VHM-Health Exam sub-cohort with complete records of those two covariables (n = 89,362). This was because baseline age, follow-up times, and sex ratios were very similar in the matched health examination sub-cohort with complete records for occupational status and BMI (n = 25,691) (Table S1).

### 2.4. Outcome

The Cancer Registry Vorarlberg provided data on melanoma deaths (ICD-10 C43), incident diagnoses of invasive melanoma (ICD-10 C43) and melanoma in situ (ICD-10 D03), as well as information on tumor characteristics of invasive melanoma, i.e., Breslow tumor thickness [mm], melanoma depth according to Clark’s level (levels 1–5), and tumor size and extension according to the T category of the TNM classification (T1–T4). Record linkage was used for identification of cases in the study cohorts. Breslow tumor thickness and Clark’s level of invasive diagnoses were analyzed in VHM-Skin vs. comparison cohorts over the entire follow-up interval up to 31 December 2019. T stage was omitted from these analyses because of changed diagnostic criteria with increased category thresholds of malignant melanoma from 2003 [30]. Because differences in tumor characteristics at diagnosis were suspected in participants during and after the program, a supplementary analysis was conducted comprising only diagnoses in VHM-Skin during the screening period (1989–1994). In this analysis, diagnoses of non-participants (i.e., in VHM-Health Exam and GP-CC) were included up to 31 December 2002, because information on tumor characteristics amongst non-participants during the screening period was sparse since more tumors were detected in the context of the skin cancer program, thus precluding comparison of equal time intervals between 1989 and 1994. On the other hand, the observation interval of the comparison groups was not extended beyond 2002 (e.g., to 2019) because of a potential bias due to imbalanced age structures due to very different observation intervals, and because of the aforementioned changes in diagnostic criteria of T staging of malignant melanoma in 2003 [30].

### 2.5. Statistical Analysis

Incidence and mortality rates were obtained by relating diagnoses of and deaths by melanoma to the average annual population of Vorarlberg retrieved from the Statistics Austria database [31] or to person-years of the skin cancer program. Poisson or negative binomial regression models were applied to assess temporal changes in age-standardized incidence rate ratios (ΔIRRs) for melanoma incidence and mortality in 5-year intervals during 1985–2019 in the entire population and to compare incidence rates of VHM-Skin with the GP-CC. Goodness of fit of the regression models was estimated by means of deviation from equidispersion as assessed from the ratio of deviance to degrees of freedom (df) and the Pearson´s *χ*^2^ to df ratio, as well as by using the Akaike and Bayesian Information Criteria (AIC and BIC, respectively). For any one evaluation, the model with deviance/df and *χ*^2^/df ratios closest to 1 and the lowest AIC and BIC values was selected. Risk evaluation in cohorts with individual-level data was conducted using the Cox regression analysis. Participants were censored on the last day of the study (31 December 2019) or at the date of their death (other than due to melanoma in analyses where melanoma death was the outcome) obtained from the mortality registry of the Statistics Austria database [31], whichever date came first. Quade´s Ancova as a non-parametric method was applied for evaluation of Breslow tumor thickness data that followed a right-skewed distribution. Ordinally scaled Clark’s level stage and T stage were evaluated using ordinal probit or ordinal logit regression analyses, whichever model showed a better fit to the data as monitored by the ratio of deviance to degrees of freedom (df) and the Pearson’s *χ*^2^ to df ratio, as well as the AIC and BIC. Shapiro–Wilk and Kolmogorov–Smirnov tests served as checks for the normality of distributions. Results were considered statistically significant at the 95% confidence level. All analyses were conducted using IBM SPSS Statistics, version 28.0 (IBM Corp., Armonk, NY, USA).

## 3. Results

Characterization of the study populations with individual-level data showed that 61.4% and 55.4% of participants of VHM-Skin and VHM-Health Exam, respectively, were women (Table 1 and Table S1). The average age was 40 years in VHM-Skin and almost 45 years in VHM-Health Exam. Median follow-up (until death or end of study) was nevertheless longer in the VHM-Health Exam group (roughly 28 years vs. 27 years) because participation peaked in the two final years of the program (1993 and 1994), while health examination visits were relatively uniformly distributed during 1989–1994. Overall death rates throughout the study time were lowest in VHM-Skin (16.0% overall, 13.9% in women, 19.2% in men). They were highest in VHM-Health Exam (26.1% overall, 24.5% in women, 28.1% in men), and also higher in the matched VHM-Health Exam sample (18.5% overall, 15.6% in women, 23.2% in men) compared to the VHM-Skin group. In VHM-Skin, the proportion of blue-collar workers (20.1%) was lower and that of white-collar workers (71.9%) higher than in VHM-Health Exam (36.6% blue- and 54.4% white-collar workers). BMI was lower in VHM-Skin (23.7 ± 3.8 kg/m^2^) than in VHM-Health Exam (25.0 ± 4.2 kg/m^2^). Moreover, 4381/8997 (48.7%) participants in VHM-Skin had attended the health examination prior to the skin cancer examination between 1 August 1989 and 31 December 1994, and 3024 of them (33.6%) were classified as high-risk patients for skin cancer.

Table S2 summarizes melanoma cases and deaths, as well as population size by gender, in the general population of Vorarlberg for every year between 1985 and 2019. In total, 2463 invasive melanomas, 1492 melanomas in situ, and 393 melanoma deaths were recorded in Vorarlberg during 1985–2019.

In the general population, age-standardized melanoma incidence significantly increased between 1985 and 2019 with respect to both invasive and in situ cancers (by 18% and 32%, respectively, for every five-year interval and for both sexes combined) (Table 2 and Table 3, Figure S1). Confined to 2000–2019, a significant rise was observed only for in situ melanomas (by 13% for every five-year interval and for both sexes combined). Melanoma mortality was generally higher in men and dropped overall from 3.56/100,000 (1985–1989) to 2.63/100,000 (2015–2019), barely missing statistical significance (ΔIRR 0.96, 95%-CI 0.91–1.01). In detail, melanoma mortality decreased non-significantly from 1985–1989 (3.56/100,000) until 2000–2004 (2.64/100,000) (ΔIRR 0.90, 95%-CI 0.78–1.03), and from 1990–1994 (3.59/100,000) until 2000–2004, followed by an increase to 3.35/100,000 until 2005–2009, and a further non-significant decrease to 2.63/100,000 until 2015–2019 (ΔIRR 0.90, 95%-CI 0.76–1.06).

Incidence rates of total invasive and total in situ melanomas diagnosed until 2019 were elevated in VHM-Skin vs. the GP-CC (Table 4). A total of 207 invasive melanoma and 187 melanoma in situ diagnoses were documented during 232,524 py in the VHM-Skin group compared to 1734 invasive melanoma and 1106 melanoma in situ diagnoses during 5,521,093 py, giving rise to incidence rate ratios of 2.92 (95%-CI 2.49–3.41) and 4.13 (95%-CI 3.53–4.83), respectively. IRRs of invasive melanoma were higher in men, but higher in women for melanoma in situ. Melanoma-specific mortality was significantly higher in the VHM-Skin group (IRR 1.66, 95%-CI 1.00–2.75; 16 vs. 250 deaths). A gender-wise evaluation revealed a slightly increased mortality in both genders without reaching statistical significance.

Incidence rates of invasive and in situ melanomas were likewise elevated in VHM-Skin relative to VHM-Health Exam (Table 5). For full follow-up until 2019, HRs were 3.02 (95%-CI 2.59–3.52) for invasive melanoma (207 diagnoses/8997 participants of VHM-Skin vs. 790 diagnoses/92,760 participants of VHM-Health Exam) and 3.90 (95%-CI 3.30–4.61) for melanoma in situ (187 diagnoses/8997 vs. 562 diagnoses/92,760). Moreover, different time intervals from baseline, i.e., up to the end of the program (31 December 1994), up to 10 years, 10–20 years, and 20+ years, demonstrated the highest HRs up to 31 December 1994, declining the more distant in the past the study entry was. For the period coinciding with the skin cancer program, HR for invasive melanoma diagnosis was as high as 18.50 (95%-CI 10.92–31.33). Additional adjustment for occupational status and BMI did not substantially alter the results, but, in general, occupational status had a stronger attenuating effect on HRs (Table S3). Consistent with the findings comparing VHM-Skin with the GP-CC, HRs for incident invasive melanoma were higher in men, although higher in women with respect to incident melanoma in situ. Also, melanoma mortality was significantly elevated in the VHM-Skin group (Table 5) (HR 2.12, 95%-CI 1.25–3.61; 16 melanoma deaths/8997 participants of VHM-Skin vs. 97 deaths/92,760 participants of VHM-Health Exam), also upon inclusion of all covariates (Table S3), and HRs decreased toward the end of the study period (Table 5). Gender-wise, a significantly higher mortality was observed in women (Table 5).

In a sensitivity analysis, the comparison between VHM-Skin and the matched VHM-Health Exam revealed similar findings (Table S4). During full follow-up, HRs were 2.82 (95%-CI 2.33–3.41) for invasive melanoma and 3.73 (95%-CI 3.01–4.62) for melanoma in situ. Invasive melanoma incidence was higher in men, and melanoma in situ incidence was higher in women in the VHM-Skin group. Melanoma mortality was significantly higher in VHM-Skin without additional adjustment for occupational status and BMI (HR 2.24, 95%-CI 1.17–4.29), and, gender-wise, only in women both without and with adjustment for additional covariates.

Finally, tumor thickness according to Breslow as well as Clark’s level at diagnosis of invasive melanoma (C43) until 2019 were significantly higher in both sexes combined and in men but not in women in the VHM-Health Exam and the GP-CC groups relative to the VHM-Skin cohort (Table 6; for results of the complete evaluation including additional covariates and the matched VHM-Health Exam cohort, cf. Table S5). When, however, only invasive melanomas detected during the screening period 1989–1994 were considered and compared with the comparison cohorts until 2002, Breslow thickness, Clark’s level, and T stage were equal or higher in VHM-Skin, albeit without attaining statistical significance (Table S6).

## 4. Discussion

We herein report results of a thorough evaluation of a population-based targeted screening program for skin cancer in western Austria between 1989 and 1994 with a median follow-up time of 27 years until 2019. Key findings include the increase in incidence of melanoma diagnoses and the higher or unchanged melanoma mortality in participants to the program. In the general population, melanoma mortality declined, though not statistically significantly, following termination of the program, as well as during the entire study period up to 2019.

Higher or unchanged melanoma mortality in the VHM-Skin group relative to the comparison groups devoid of skin cancer screening suggests that a larger proportion of individuals at high risk for melanoma were referred to the skin cancer program. In fact, a third of the participants were classified as high-risk individuals for skin cancer, which was stated to be a multiple of the general population [25]. This is also reflected by equal-to-(statistically non-significant)-more-advanced tumor characteristics of invasive melanomas in VHM-Skin until 1994 relative to the comparison cohorts until 2002, since less advanced tumor characteristics should be expected in a non-targeted screening approach. Similarly, a recent evaluation of the German skin cancer screening reported higher screening prevalence in patients who eventually died of melanoma relative to the matched control patients, suggesting inclusion of examinations not for screening purposes [32]. This finding was traced to insurance billing codes for screening that are used also for not occasion-free examinations. In our study, however, examination protocols of the program were instead obtained from the physicians, and examinations not conducted for screening purposes were billed differently, ensuring that only screening patients entered the program.

Our observation that the relative risk of invasive melanoma detection in the VHM-Skin group relative to the comparison groups was higher in men than in women and that tumor characteristics tended to be less advanced in women even as non-participants to the skin cancer program may be reflective of women´s generally more careful and informed attitude toward skin health [33]. Indeed, the small amount of melanoma deaths in female comparison cohorts underlies the significantly higher melanoma mortality in women in the VHM-Skin group. Documentation of elevated Breslow thickness and Clark’s level in men but not in women in the comparison groups is consistent with this finding, assuming that the VHM-Skin participants with lower melanoma risk continued to visit their dermatologists after cessation of the program, leading to detection of predominantly low-stage melanomas. Accordingly, incidence of melanoma diagnoses in VHM-Skin was higher relative to the comparison groups even after 1994. Furthermore, we found that risk of dying from melanoma in the VHM-Skin group was less distinctly elevated in both sexes combined and women compared to the GP-CC and the VHM-Health Exam groups. A more health-conscious behavior among VHM-Health Exam participants compared with the general population, especially in women, might explain this finding.

Moreover, results herein portend additional presence of health screen bias in the VHM-Skin group. First, overall mortality was lower than in the matched VHM-Health Exam sample. Consistently, in attendees of the German skin cancer screening program, overall mortality was found to be lower than that recorded among non-participants [34]. Second, participants in the program between 1989 and 1994 were at higher risk of melanoma diagnosis than individuals in the comparison cohorts, even after 1994. Tumor stages in the VHM-Skin group including diagnoses until 2019 were less advanced than melanomas detected during the screening period (1989–1994) only. Hence, diagnoses of lower stage melanomas prevailed among screening participants after termination of the program, a phenomenon which could be the result of regular dermatological visits by health-conscious patients. Combined findings thus demonstrate a mixed risk profile in the skin cancer program.

Following termination of the skin cancer screening program, a marked but statistically non-significant reduction of melanoma mortality was observed until 2004. Even though this is the expected time frame for a potential contribution of the program to mortality reduction, the VHM-Skin cohort was very small relative to the size of the general population at that time (below 3%). Moreover, the reduction failed to reach statistical significance, a result which could indicate chance oscillation. On the other hand, in a previously described targeted screening setting based on self-selection of high-risk individuals, the number of patients needed to screen to find one more melanoma case was estimated to be 11 times lower than using a non-targeted strategy [35], and relative melanoma risk was assessed to rise roughly 14-fold relative to individuals not at high risk [36]. Similarly, in our analysis, the hazard of an invasive melanoma diagnosis was increased 18.5-fold in the VHM-Skin relative to the VHM-Health Exam cohort during the screening period 1989–1994. Hence, a targeted approach could be a successful way to assemble vulnerable individuals who benefit the most from timely intervention. In Vorarlberg, the participation rate in the health examination program was very high [23,24], ensuring that a large proportion of the general population received a preliminary check-up of the skin which effectuated the pre-selection of a cohort at high-risk for melanoma. Finally, for the other statistically non-significant downward trend of melanoma mortality after 2009, a plausible cause can be proposed, namely a benefit from novel immunotherapy for treating advanced melanomas [37,38]. This decline has also been observed in Germany since 2014 and has been ascribed to new immune-therapeutic options rather than to effects of the national German skin cancer screening implemented in 2008 [39]. This could be regarded as an argument for a real, non-random effect in our data also before 2005. In Vorarlberg, melanoma mortality reduction in the whole study period of 1985–2019, which just barely missed statistical significance, could thus be the result of both interventions during two different time intervals.

At the population level, skin cancer screening effects on melanoma mortality have not been conclusively demonstrated. In the German province of Schleswig-Holstein, melanoma mortality in 2008 and 2009 exhibited a decrease of 49% and 45% in women and men, respectively, compared to 1998/1999 following screening in 2003 and 2004 in which 19% of the adult population participated [40]. A subsequent analysis, however, revealed that mortality rates started to rebound steeply already in 2009 with regard to men and in 2011 with regard to women and continued at the levels of 1998/1999 after 2011 [16]. The decrease in melanoma mortality characterizing the preceding years could have thus been due to random fluctuations and/or coding irregularities for the causes of death from 2007 to 2010 [16,41]. Beyond that, the utility of the nationwide melanoma screening program introduced in Germany in 2008 for the population ≥35 years remains questionable to date, since several evaluation studies failed to observe a correlation with melanoma mortality [16,17,18,19] even in region-specific analyses [18] and when only early melanoma stages were considered [19]. However, all-cause mortality was lower in melanoma patients who had undergone screening compared to those who had not undergone screening [34], analogously to our study. By contrast, a non-population-based investigation in employees of a large research laboratory in California participating in a screening program showed a clear reduction in melanoma-related deaths [15].

This study has a number of limitations, but also strengths. In Austria, the legal obligation to report cancer cases to the cancer registries is restricted to hospitals. Therefore, records of melanomas that are frequently diagnosed and treated by practicing dermatologists are incomplete [42]. Varying or increasing numbers of melanoma cases since 1985 (Table S2) must be primarily regarded as the result of improved completeness of documentation. The rise in melanoma diagnoses in 1993 mirrors the beginning of systematic inquiries into the reporting by physicians and hospital departments by the Cancer Registry Vorarlberg. A further rise in 2002 coincides with the establishment of a new dermatological department in the largest hospital of Vorarlberg, i.e., Landeskrankenhaus Feldkirch, and 2004 marked the year that electronic data transfer from reporters to the local cancer registry was introduced, entailing access to new data sources from pathology institutes. In this regard, under-identification of prevalent cases from 1985 until study entry and of invasive and in situ diagnoses until the end of follow-up cannot be ruled out. If incompleteness of cases were evenly distributed across all cohorts, this potential bias should, however, be limited. Despite incomplete documentation of diagnoses, we deem incidence rates and hazard ratios to be reliable, based on plausible results herein. Moreover, a high percentage of case completeness for invasive melanoma diagnoses has been reported previously for western Austria including Vorarlberg [42]. Likewise, information on all tumor characteristics at invasive melanoma diagnosis was not available for every patient. However, documentation of deaths due to melanoma was virtually complete. Next, we had no detailed information on the individual risk variables at the screening examination, and the proportion of high-risk individuals in the comparison groups could not be assessed. Also, we cannot exclude bias due to loss to follow-up. However, the geographic location as well as a different health insurance system in the neighboring non-EU countries Switzerland and Liechtenstein impede medical treatment to be utilized outside of Vorarlberg. Finally, even though a drop in melanoma mortality in the general population was observed in the years following the termination of the program, no conclusions regarding causality can be drawn based on our results.

On the other hand, strengths include the clear identification of screening examinations as such and the direct link with the examination protocols obtained from the physicians based on electronic data collection, the availability of individual-level data for screening and comparison cohorts which facilitated the investigation of potentially relevant covariates, and the extensive follow-up of up to almost three decades.

## 5. Conclusions

In a population-based screening program in Western Austria, we identified individuals at high risk of developing melanoma. The increase in both melanoma incidence and mortality relative to the comparison groups indicates successful implementation of a targeted screening approach. Focusing efforts on high-risk populations as in a targeted screening could be a promising alternative to costly mass screening with questionable effectiveness.

## Figures and Tables

**Table 1:** Characteristics of VHM-Skin and VHM-Health Exam participants.

			VHM-Skin	VHM-Health Exam
All				
	n		8997	92,760
	Age at baseline examination (years), mean ± SD		40.0 ± 15.0	44.8 ± 15.3
	Follow-up * (years), median (IQR)		26.8 (25.8–28.5)	27.8 (25.4–29.3)
	Overall deaths, n (%)		1438 (16.0%)	24,210 (26.1%)
Women				
	n		5528	51,412
	Age at baseline examination (years), mean ± SD		39.4 ± 14.9	45.2 ± 15.8
	Follow-up * (years), median (IQR)		26.9 (25.8–28.6)	28.0 (25.6–29.4)
	Overall deaths, n (%)		771 (13.9%)	12,602 (24.5%)
Men				
	n		3469	41,348
	Age at baseline examination (years), mean ± SD		40.8 ± 15.2	44.3 ± 14.8
	Follow-up * (years), median (IQR)		26.7 (25.6–28.4)	27.6 (25.2–29.2)
	Overall deaths, n (%)		667 (19.2%)	11,608 (28.1%)

* follow-up until death or end of study.

**Table 2:** Average annual incidence of invasive melanoma and melanoma in situ, and average annual melanoma mortality in 5-year intervals between 1985 and 2019 by gender, age-standardized for the population of Vorarlberg between 1985 and 2019.

		1985–1989	1990–1994	1995–1999	2000–2004	2005–2009	2010–2014	2015–2019
All								
	Invasive melanoma (C43)	8.10	12.52	14.92	23.11	27.27	24.84	20.61
	Melanoma in situ (D03)	1.04	6.42	9.31	12.00	13.89	18.35	16.56
	Melanoma mortality	3.56	3.59	3.16	2.64	3.35	3.23	2.63
Women								
	Invasive melanoma (C43)	9.65	11.92	15.92	21.28	24.05	24.22	19.35
	Melanoma in situ (D03)	1.44	7.00	11.38	13.09	14.11	18.90	15.64
	Melanoma mortality	3.83	2.77	3.19	2.41	2.82	2.80	2.54
Men								
	Invasive melanoma (C43)	6.50	13.13	13.89	25.00	30.58	25.47	21.90
	Melanoma in situ (D03)	0.64	5.83	7.18	10.88	13.66	17.78	17.50
	Melanoma mortality	3.28	4.44	3.13	2.88	3.90	3.67	2.72

**Table 3:** ΔIRR (incidence rate ratio change) across 5-year intervals of average annual incidence of invasive melanoma and melanoma in situ, and average annual melanoma mortality for indicated time intervals by gender, Vorarlberg Province, Austria, 1989–2019.

		ΔIRR * per 5-Year Intervals (95%-CI)			
		(1985–1990)– (2015–2019)	(2000–2004)– (2015–2019)	(1985–1990)– (2000–2004)	(2005–2009)– (2015–2019)
All					
	Invasive melanoma (C43)	1.18 (1.13–1.22)	0.96 (0.90–1.03)	-	-
	Melanoma in situ (D03)	1.32 (1.26–1.38)	1.13 (1.07–1.20)	-	-
	Melanoma mortality	0.96 (0.91–1.01)	0.99 (0.88–1.11)	0.90 (0.78–1.03)	0.90 (0.76–1.06)
Women					
	Invasive melanoma (C43)	1.13 (1.09–1.18)	0.97 (0.91–1.03)	-	-
	Melanoma in situ (D03)	1.24 (1.19–1.29)	1.08 (1.01–1.16)	-	-
	Melanoma mortality	0.95 (0.89–1.03)	1.01 (0.85–1.20)	0.88 (0.73–1.06)	0.96 (0.74–1.24)
Men					
	Invasive melanoma (C43)	1.19 (1.13–1.26)	0.94 (0.89–1.01)	-	-
	Melanoma in situ (D03)	1.35 (1.28–1.44)	1.18 (1.08–1.27)	-	-
	Melanoma mortality	0.96 (0.89–1.04)	0.97 (0.83–1.12)	0.92 (0.72–1.17)	0.85 (0.68–1.07)

* Incidence rate ratios (IRR) were adjusted for sex and age group.

**Table 4:** Incident diagnoses and incidence rates (IRs) for invasive melanoma and melanoma in situ, number of melanoma deaths and mortality rates (MRs), and incidence rate ratios (IRRs) in VHM-Skin vs. GP-CC by gender, Vorarlberg Province, Austria, 1989–2019.

		VHM-Skin			GP-CC			IRR ** (95%-CI)
		n	Person-Years	IR */MR *	n	Person-Years	IR */MR *	
Invasive melanoma (C43)								
	All	207	232,524	89	1734	5,521,093	31.4	2.92 (2.49–3.41)
	Women	94	144,309	65.1	789	2,826,810	27.9	2.40 (1.93–2.98)
	Men	112	88,215	127	945	2,694,283	35.1	3.38 (2.77–4.12)
Melanoma in situ (D03)								
	All	187	232,524	80.4	1106	5,521,093	20	4.13 (3.53–4.83)
	Women	115	144,309	79.7	538	2,826,810	19	4.43 (3.61–5.43)
	Men	71	88,215	80.5	568	2,694,283	21.1	3.67 (2.86–4.71)
Melanoma deaths								
	All	16	232,524	6.9	250	5,521,093	4.5	1.66 (1.00–2.75)
	Women	8	144,309	5.5	112	2,826,810	4	1.64 (0.80–3.37)
	Men	8	88,215	9.1	138	2,694,283	5.1	1.64 (0.80–3.34)

* Incidence and mortality rates (IR, MR) were unadjusted. ** Incidence rate ratios (IRR) were adjusted for sex, age group, and time period.

**Table 5:** Hazard ratios (HRs) for incident invasive melanoma and melanoma in situ diagnoses as well as melanoma deaths in VHM-Skin vs. VHM-Health Exam as reference for follow-up intervals as indicated by gender, Vorarlberg Province, Austria, 1989–2019. N, individuals in VHM-Health Exam; n, individuals in VHM-Skin.

			Full Follow-Up	Follow-Up until 31 December 1994	0–10 Years	>10–20 Years	20+ Years
Incident invasive melanoma (C43)							
	All						
		(sub)cohort size, n/N	8997/92,760	8997/92,760	8997/92,760	8644/87,072	8045/78,123
		cases, n/N	207/790	30/27	61/165	99/338	47/287
		HR (95%-CI) *	3.02 (2.59–3.52)	18.50 (10.92–31.33)	4.33 (3.22–5.82)	3.27 (2.61–4.10)	1.94 (1.42–2.65)
	Women						
		(sub)cohort size, n/N	5528/51,412	5528/51,412	5528/51,412	5367/48,754	5053/44,076
		cases, n/N	95/371	14/12	29/91	45/152	21/128
		HR (95%-CI) **	2.55 (2.03–3.21)	19.14 (8.75–41.87)	3.44 (2.26–5.25)	2.87 (2.05–4.02)	1.59 (1.00–2.53)
	Men						
		(sub)cohort size, n/N	3469/41,348	3469/41,348	3469/41,348	3277/38,318	2992/34,047
		cases, n/N	112/419	16/15	32/74	54/186	26/159
		HR (95%-CI) **	3.46 (2.81–4.27)	18.80 (9.21–38.39)	5.56 (3.67–8.43)	3.61 (2.67–4.90)	2.24 (1.48–3.40)
Incident melanoma in situ (D03)							
	All						
		(sub)cohort size, n/N	8997/92,760	8997/92,760	8997/92,760	8653/87,123	8066/78,235
		cases, n/N	187/562	17/23	50/98	80/231	57/233
		HR (95%-CI) *	3.90 (3.30–4.61)	12.76 (6.74–24.16)	6.22 (4.41–8.77)	3.78 (2.93–4.88)	3.04 (2.27–4.07)
	Women						
		(sub)cohort size, n/N	5528/51,412	5528/51,412	5528/51,412	5360/48,782	5041/44,127
		cases, n/N	115/276	9/13	33/56	49/110	33/110
		HR (95%-CI) **	4.37 (3.50–5.44)	10.78 (4.52–25.74)	6.77 (4.38–10.46)	4.27 (3.04–6.01)	3.26 (2.19–4.83)
	Men						
		(sub)cohort size, n/N	3469/41,348	3469/41,348	3469/41,348	3293/38,341	3025/34,108
		cases, n/N	72/286	8/10	17/42	31/121	24/123
		HR (95%-CI) **	3.32 (2.56–4.30)	15.35 (5.98–39.40)	5.37 (3.06–9.45)	3.16 (2.13–4.70)	2.75 (1.77–4.26)
Melanoma deaths							
	All						
		(sub)cohort size, n/N	8997/92,760	8997/92,760	8997/92,760	8701/87,214	8182/78,531
		cases, n/N	16/97	2/4	4/20	8/37	4/40
		HR (95%-CI) *	2.12 (1.25–3.61)	10.40 (1.88–57.64)	2.62 (0.89–7.67)	2.61 (1.21–5.62)	1.36 (0.49–3.82)
	Women						
		(sub)cohort size, n/N	5528/51,412	-	-	-	-
		cases, n/N	8/38	-	-	-	-
		HR (95%-CI) **	2.49 (1.16–5.37)	-	-	-	-
	Men						
		(sub)cohort size, n/N	3469/41,348	-	-	-	-
		cases, n/N	8/59	-	-	-	-
		HR (95%-CI) **	1.83 (0.87–3.83)	-	-	-	-

* Adjusted for baseline age and sex, ** adjusted for baseline age.

**Table 6:** Breslow thickness and Clark’s level at diagnosis of invasive melanoma throughout the study period 1989–2019 in VHM-Skin vs. VHM-Health Exam and vs. the GP-CC by gender. IQR, interquartile range; SD, standard deviation.

		VHM-Skin		VHM-Health Exam			GP-CC		
Breslow thickness (mm)									
		n	median (IQR)	n	median (IQR)	p *	n	median (IQR)	p *
	All	185	0.5 (0.3–0.8)	652	0.6 (0.4–1.3)	<0.05	1443	0.6 (0.4–1.4)	<0.01
	Women	84	0.5 (0.3–0.8)	298	0.6 (0.4–1.2)	0.29	631	0.6 (0.4–1.4)	0.11
	Men	101	0.5 (0.3–0.8)	354	0.6 (0.4–1.3)	<0.05	812	0.7 (0.4–1.5)	<0.05
Clark´s level (1–5)									
		n	mean (±SD)	n	mean (±SD)	p *	n	mean (±SD)	p *
	All	176	3.0 ± 0.7	630	3.1 ± 0.8	<0.05	1368	3.2 ± 0.9	<0.01
	Women	81	3.0 ± 0.8	295	3.1 ± 0.8	0.36	744	3.1 ± 0.9	0.11
	Men	95	3.0 ± 0.6	335	3.1 ± 0.9	<0.05	624	3.2 ± 0.8	<0.01

* adjusted for baseline age, and sex (all).

## Data Availability

Data supporting the findings of this study are available from the corresponding author upon reasonable request.

## Supplementary Materials

The following supporting information can be downloaded at: https://www.mdpi.com/article/10.3390/cancers16071283/s1, Table S1: Characteristics of VHM-Skin, VHM-Health Exam, and *matched* VHM-Health Exam participants; Table S2: Yearly melanoma cases and deaths, and annual average population size in Vorarlberg 1995–2019; Table S3: Hazard ratios for incident invasive melanoma and melanoma in situ diagnoses as well as melanoma deaths in VHM-Skin vs. VHM-Health Exam as reference for follow-up intervals as indicated by gender, including occupational status and BMI as covariates; Table S4: Hazard ratios for incident invasive melanoma and melanoma in situ diagnoses as well as melanoma deaths in VHM-Skin vs. *matched* VHM-Health Exam as reference for follow-up intervals as indicated by gender, not including (upper part) and including (lower part) occupational status and BMI as covariates; Table S5: Breslow thickness and Clark’s level at diagnosis of invasive melanoma throughout the study period 1989–2019 in VHM-Skin vs. VHM-Health Exam, vs. *matched* VHM-Health Exam, and vs. the GP-CC by gender; Table S6: Breslow thickness, Clark’s level, and T score at diagnosis of invasive melanoma in VHM-Skin (1989–1994) vs. VHM-Health Exam (1989–2002), vs. *matched* VHM-Health Exam (1989–2002), and vs. the GP-CC (1993–2002) by gender; Figure S1: Line graphs showing trends of age-adjusted invasive melanoma incidence, melanoma in situ incidence, and melanoma mortality in the general population of Vorarlberg in 5-year intervals 1989–2019.
