# Supplementary material for: Evaluation of a Population-Based Targeted Screening Approach for Skin Cancer with Long-Time Follow-Up in Austria including Potential Effects on Melanoma Mortality
Source: Cancers (Basel). 2024 Mar 26;16(7):1283. doi: 10.3390/cancers16071283 (PMC11011036; doi:10.3390/cancers16071283)
Supplement: Supplementary file 1 [file cancers-16-01283-s001.zip › cancers-2867882-supplementary.pdf]

Table S1: Characteristics of VHM-Skin, VHM-Health Exam, and *matched* VHM-Health Exam participants.

	**VHM-Skin**		**VHM-Health Exam**		***matched* VHM-Health Exam**	
	**Full cohort**	**Sub-cohort (all covariates)**	**Full cohort**	**Sub-cohort (all covariates)**	**Full cohort**	**Sub-cohort (all covariates)**
**All**						
n	8997	7543	92,760	89,362	26,991	25,691
Age at baseline examination (years), mean ± SD	40.0 ± 15.0	40.7 ± 14.6	44.8 ± 15.3	44.9 ± 15.3	40.0 ± 15.0	40.3 ± 15.0
Follow-up* (years), median (IQR)	26.8 (25.8 - 28.5)	26.9 (25.8 - 28.6)	27.8 (25.4 - 29.3)	27.9 (25.4 - 29.3)	26.8 (25.5 - 28.5)	26.8 (25.6 - 28.5)
overall deaths, n (%)	1438 (16.0%)	1193 (15.8%)	24,210 (26.1%)	23,440 (26.2%)	4994 (18.5%)	4808 (18.7%)
Occupational status, *n* (%)	-		-		-	
blue collar		1514 (20.1%)		32,666 (36.6%)		8925 (34.8%)
white collar		5422 (71.9%)		48,659 (54.4%)		14,266 (55.5%)
self-employed		607 (8.0%)		8037 (9.0%)		2500 (9.7%)
BMI, mean ± SD	-	23.7 ± 3.8	-	25.0 ± 4.2	-	24.5 ± 4.4
**Women**						
n	5528	4687	51,412	49,736	16,584	15,905
Age at baseline examination (years), mean ± SD	39.4 ± 14.9	40.0 ± 14.4	45.2 ± 15.8	45.3 ± 15.7	39.5 ± 14.9	39.6 ± 14.9
Follow-up* (years), median (IQR)	26.9 (25.8 - 28.6)	27.0 (25.9 - 28.6)	28.0 (25.6 - 29.4)	28.1 (25.7 - 29.4)	27.0 (25.6 - 28.6)	27.0 (25.7 - 28.6)
overall deaths, n (%)	771 (13.9%)	638 (13.6%)	12,602 (24.5%)	12,185 (24.5%)	2584 (15.6%)	2493 (15.7%)
Occupational status, *n* (%)	-		-		-	
blue collar		1023 (21.8%)		18,768 (37.7%)		5367 (33.7%)
white collar		3316 (70.8%)		27,182 (54.7%)		9128 (57.4%)
self-employed		348 (7.4%)		3786 (7.6%)		1410 (8.9%)
BMI, mean ± SD	-	23.0 ± 4.0	-	24.6 ± 4.6	-	24.1 ± 4.7
**Men**						
n	3469	2856	41,348	39,626	10,407	9786
Age at baseline examination (years), mean ± SD	40.8 ± 15.2	41.8 ± 14.8	44.3 ± 14.8	44.6 ± 14.7	40.8 ± 15.2	41.3 ± 15.2
Follow-up* (years), median (IQR)	26.7 (25.6 - 28.4)	26.8 (25.6 - 28.4)	27.6 (25.2 - 29.2)	27.7 (25.1 - 29.2)	26.7 (25.3 - 28.3)	26.7 (25.3 - 28.3)
overall deaths, n (%)	667 (19.2%)	555 (19.4%)	11,608 (28.1%)	11,255 (28.4%)	2410 (23.2%)	2315 (23.7%)
Occupational status, *n* (%)	-		-		-	
blue collar		491 (17.2%)		13,898 (35.1%)		3558 (36.4%)
white collar		2106 (73.7%)		21,477 (54.2%)		5138 (52.5%)
self-employed		259 (9.1%)		4251 (10.7%)		1090 (11.1%)
BMI, mean ± SD	-	24.8 ± 3.1	-	25.5 ± 3.5	-	25.2 ± 3.6

* follow-up until death or end of study

Table S2: Yearly melanoma cases and deaths, and annual average population size in Vorarlberg 1995-2019.

Year	New invasive melanoma diagnoses (C43)			New diagnoses of melanoma *in situ* (D03)			Deaths due to melanoma			Annual average population		
	All	Women	Men	All	Women	Men	All	Women	Men	All	Women	Men
**1985**	23	18	5	4	3	1	9	3	6	309 043	158 094	150 949
**1986**	27	19	8	5	4	1	6	3	3	311 237	159 089	152 148
**1987**	26	12	14	1	1	0	9	8	1	313 196	160 000	153 196
**1988**	34	19	15	4	2	2	12	6	6	315 419	161 072	154 347
**1989**	27	15	12	5	5	0	10	6	4	320 470	163 423	157 047
**1990**	24	13	11	10	6	4	10	5	5	326 594	166 083	160 511
**1991**	33	17	16	7	4	3	11	7	4	331 930	168 354	163 576
**1992**	31	15	16	8	4	4	10	2	8	336 160	170 298	165 862
**1993**	58	31	27	37	24	13	8	5	3	338 640	171 428	167 212
**1994**	38	16	22	33	16	17	12	2	10	340 471	172 240	168 231
**1995**	47	26	21	30	18	12	4	4	0	341 951	173 033	168 918
**1996**	42	22	20	25	17	8	11	5	6	343 135	173 787	169 348
**1997**	60	27	33	35	20	15	8	4	4	344 354	174 499	169 855
**1998**	50	33	17	34	20	14	12	5	7	345 766	175 266	170 500
**1999**	43	25	18	24	18	6	14	7	7	347 443	176 101	171 342
**2000**	68	40	28	30	18	12	4	1	3	349 257	177 011	172 246
**2001**	56	30	26	24	13	11	9	4	5	351 356	177 980	173 376
**2002**	76	33	43	38	18	20	12	5	7	353 635	179 083	174 552
**2003**	77	32	45	52	29	23	7	5	2	355 621	180 123	175 498
**2004**	126	54	72	65	38	27	13	6	7	358 521	181 654	176 867
**2005**	121	52	69	50	23	27	13	3	10	361 391	183 113	178 278
**2006**	111	49	62	50	29	21	10	8	2	363 389	184 021	179 368
**2007**	82	31	51	50	31	19	16	5	11	364 985	184 977	180 008
**2008**	95	38	57	50	23	27	13	7	6	366 397	185 877	180 520
**2009**	109	58	51	64	29	35	12	4	8	367 590	186 569	181 021
**2010**	121	52	69	92	50	42	18	11	7	368 894	187 204	181 690
**2011**	95	46	49	99	46	53	12	5	7	370 096	187 901	182 195
**2012**	100	48	52	65	25	40	18	8	10	371 697	188 727	182 970
**2013**	104	56	48	62	30	32	10	2	8	373 870	189 642	184 228
**2014**	92	42	50	59	40	19	13	4	9	376 973	191 076	185 897
**2015**	109	55	54	51	26	25	16	8	8	381 000	192 650	188 350
**2016**	76	35	41	56	31	25	11	5	6	386 708	194 961	191 747
**2017**	97	37	60	76	28	48	14	7	7	390 296	196 730	193 566
**2018**	94	47	47	114	45	69	12	4	8	393 025	197 996	195 029
**2019**	91	36	55	83	42	41	14	6	8	395 949	199 401	196 548
**Σ**	**2463**	**1179**	**1284**	**1492**	**776**	**716**	**393**	**180**	**213**			

Table S3: Hazard ratios for incident invasive melanoma and melanoma *in situ* diagnoses as well as melanoma deaths in VHM-Skin vs. VHM-Health Exam as reference for follow-up intervals as indicated by gender, including occupational status and BMI as covariates, Vorarlberg Province, Austria, 1989-2019. N, individuals in the VHM-Health Exam cohort; n, individuals in the VHM-Skin cohort

	**Incident invasive melanoma (C43)**					**Incident melanoma *in situ* (D03)**					**Melanoma deaths**
	**Full follow-up**	**Follow-up until Dec. 31st, 1994**	**0-10 years**	**>10-20 years**	**20+ years**	**Full follow-up**	**Follow-up until Dec. 31st, 1994**	**0-10 years**	**>10-20 years**	**20+ years**	**Full follow-up**
**All**											
**(sub)cohort size, n / N**	7543 / 89,362	7543 / 89,362	7543 / 89,362	7289 / 83,905	6785 / 75,222	7543 / 89,362	7543 / 89,362	7543 / 89,362	7295 / 83,955	6810 / 75,337	7543 / 89,362
**cases, n / N**	182 / 765	25 / 26	51 / 155	88 / 333	43 / 277	160 / 547	16 / 21	45 / 92	65 / 222	50 / 233	12 / 93
**HR (95%-CI)** [a]	3.07 (2.61-3.61)	18.22 (10.43-31.84)	4.34 (3.16-5.97)	3.30 (2.61-4.18)	2.06 (1.49-2.84)	3.86 (3.23-4.61)	15.32 (7.89-29.77)	6.78 (4.73-9.71)	3.60 (2.73-4.75)	2.99 (2.20-4.06)	1.84 (1.01-3.37)
**HR (95%-CI)** [b]	2.96 (2.51-3.48)	18.32 (10.37-32.36)	4.15 (3.01-5.74)	3.15 (2.48-3.99)	2.02 (1.46-2.80)	3.67 (3.07-4.39)	14.87 (7.56-29.26)	6.51 (4.52-9.37)	3.40 (2.56-4.50)	2.84 (2.08-3.88)	1.86 (1.02-3.42)
**Women**											
**(sub)cohort size, n / N**	4687 / 49,736	4687 / 49,736	4687 / 49,736	4577 / 47,199	4316 / 42,667	4687 / 49,736	4687 / 49,736	4687 / 49,736	4569 / 47,227	4309 / 42,718	4687 / 49,736
**cases, n / N**	83 / 357	10 / 11	22 / 84	41 / 150	20 / 123	98 / 270	8 / 12	29 / 52	41 / 108	28 / 110	6 / 35
**HR (95%-CI)** [c]	2.58 (2.03-3.28)	16.74 (6.97-40.21)	3.11 (1.93-4.99)	2.96 (2.09-4.19)	1.77 (1.10-2.85)	4.26 (3.37-5.38)	12.01 (4.79-30.11)	7.30 (4.60-11.56)	4.09 (2.84-5.87)	3.09 (2.03-4.70)	2.23 (0.94-5.34)
**HR (95%-CI)** [d]	2.49 (1.95-3.17)	17.06 (7.01-41.53)	2.96 (1.84-4.78)	2.81 (1.98-4.00)	1.76 (1.09-2.84)	4.12 (3.25-5.22)	10.17 (3.99-25.96)	6.84 (4.29-10.91)	4.08 (2.83-5.90)	2.94 (1.93-4.49)	2.39 (0.99-5.76)
**Men**											
**(sub)cohort size, n / N**	2856 / 39,626	2856 / 39,626	2856 / 39,626	2712 / 36,706	2469 / 32,555	2856 / 39,626	2856 / 39,626	2856 / 39,626	2726 / 36,728	2501 / 32,619	2856 / 39,626
**cases, n / N**	99 / 408	15 / 15	29 / 71	47 / 183	23 / 154	62 / 277	8 / 9	16 / 40	24 / 114	22 / 123	6 / 58
**HR (95%-CI)** [c]	3.54 (2.84-4.41)	20.05 (9.71-41.43)	5.99 (3.88-9.23)	3.60 (2.61-4.96)	2.28 (1.47-3.54)	3.33 (2.53-4.39)	19.77 (7.52-51.99)	6.01 (3.36-10.74)	2.94 (1.89-4.56)	2.82 (1.79-4.45)	1.55 (0.67-3.59)
**HR (95%-CI)** [d]	3.42 (2.74-4.27)	19.64 (9.36-41.20)	5.82 (3.75-9.05)	3.42 (2.47-4.73)	2.26 (1.45-3.51)	3.11 (2.35-4.11)	22.44 (8.40-60.00)	6.00 (3.32-10.82)	2.61 (1.67-4.07)	2.69 (1.70-4.26)	1.50 (0.64-3.49)

Adjusted for: [a] baseline age and sex; [b] baseline age, sex, occupational status, and BMI; [c] baseline age; [d] baseline age, occupational status, and BMI

Table S4: Hazard ratios for incident invasive melanoma and melanoma *in situ* diagnoses as well as melanoma deaths in VHM-Skin vs. *matched* VHM-Health Exam as reference for follow-up intervals as indicated by gender, not including (upper part) and including (lower part) occupational status and BMI as covariates, Vorarlberg Province, Austria, 1989-2019. N, individuals in the matched VHM-Health Exam cohort; n, individuals in the VHM-Skin cohort

	Incident invasive melanoma (C43)					Incident melanoma *in situ* (D03)					Melanoma deaths
	Full follow-up	Follow-up until Dec. 31st, 1994	0-10 years	>10-20 years	20+ years	Full follow-up	Follow-up until Dec. 31st, 1994	0-10 years	>10-20 years	20+ years	Full follow-up
**Matched cohorts**											
**All**											
(sub)cohort size, n / N	8997 / 26,991	8997 / 26,991	8997 / 26,991	8644 / 25,789	8045 / 23,859	8997 / 26,991	8997 / 26,991	8997 / 26,991	8653 / 25,804	8066 / 23,895	8997 / 26,991
cases, n / N	207 / 218	30 / 8	61 / 57	99 / 90	47 / 71	187 / 149	17 / 10	50 / 38	80 / 58	57 / 53	16 / 21
HR (95%-CI) [a]	2.82 (2.33-3.41)	11.27 (5.17-24.58)	3.20 (2.23-4.60)	3.24 (2.44-4.32)	1.92 (1.33-2.78)	3.73 (3.01-4.62)	5.12 (2.35-11.19)	3.94 (2.58-6.00)	4.08 (2.91-5.72)	3.13 (2.15-4.55)	2.24 (1.17-4.29)
**Women**											
(sub)cohort size, n / N	5528 / 16,584	5528 / 16,584	5528 / 16,584	5366 / 16,041	5052 / 15,039	5528 / 16,584	5528 / 16,584	5528 / 16,584	5359 / 16,048	5040 / 15,054	5528 / 16,584
cases, n / N	95 / 111	14 / 4	29 / 33	45 / 44	21 / 34	115 / 89	9 / 7	33 / 24	49 / 34	33 / 31	8 / 6
HR (95%-CI) [b]	2.55 (1.94-3.36)	10.45 (3.44-31.74)	2.63 (1.60-4.34)	3.04 (2.01-4.61)	1.83 (1.06-3.15)	3.86 (2.93-5.09)	3.87 (1.44-10.40)	4.12 (2.44-6.97)	4.30 (2.78-6.66)	3.16 (1.93-5.15)	3.96 (1.37-11.40)
**Men**											
(sub)cohort size, n / N	3469 / 10,407	3469 / 10,407	3469 / 10,407	3278 / 9748	2993 / 8820	3469 / 10,407	3469 / 10,407	3469 / 10,407	3294 / 9756	3026 / 8841	3469 / 10,407
cases, n / N	112 / 107	16 / 4	32 / 24	54 / 46	26 / 37	72 / 60	8 / 3	17 / 14	31 / 24	24 / 22	8 / 15
HR (95%-CI) [b]	3.10 (2.38-4.04)	12.09 (4.04-36.17)	3.99 (2.35-6.77)	3.43 (2.31-5.08)	1.99 (1.21-3.29)	3.53 (2.50-4.97)	8.04 (2.13-30.31)	3.63 (1.79-7.36)	3.75 (2.20-6.38)	3.07 (1.72-5.48)	1.56 (0.66-3.67)

Table S4: continued

	Incident invasive melanoma (C43)					Incident melanoma *in situ* (D03)					Melanoma deaths
	Full follow-up	Follow-up until Dec. 31st, 1994	0-10 years	>10-20 years	20+ years	Full follow-up	Follow-up until Dec. 31st, 1994	0-10 years	>10-20 years	20+ years	Full follow-up
**Matched cohorts, additional covariates**											
**All**											
**(sub)cohort size, n / N**	7543 / 25,691	7543 / 25,691	7543 / 25,691	7289 / 24,537	6785 / 22,679	7543 / 25,691	7543 / 25,691	7543 / 25,691	7295 / 24,551	6810 / 22,715	7543 / 25,691
**cases, n / N**	182 / 211	25 / 7	51 / 54	88 / 89	43 / 68	160 / 146	16 / 10	45 / 37	65 / 56	50 / 53	12 / 20
**HR (95%-CI) [c]**	2.84 (2.33-3.46)	12.17 (5.26-28.14)	3.17 (2.16-4.65)	3.26 (2.43-4.37)	2.04 (1.39-2.99)	3.58 (2.86-4.48)	5.59 (2.54-12.33)	4.08 (2.64-6.30)	3.83 (2.68-5.47)	3.02 (2.05-4.45)	1.88 (0.92-3.85)
**HR (95%-CI) [d]**	2.72 (2.22-3.33)	11.39 (4.86-26.67)	2.96 (2.00-4.37)	3.09 (2.29-4.17)	2.02 (1.37-2.98)	3.42 (2.72-4.30)	5.47 (2.45-12.23)	3.94 (2.53-6.14)	3.58 (2.49-5.16)	2.94 (1.98-4.36)	1.74 (0.84-3.61)
**Women**											
**(sub)cohort size, n / N**	4687 / 15,905	4687 / 15,905	4687 / 15,905	4577 / 15,384	4316 / 14,417	4687 / 15,905	4687 / 15,905	4687 / 15,905	4569 / 15,391	4309 / 14,432	4687 / 15,905
**cases, n / N**	83 / 108	10 / 4	22 / 32	41 / 44	20 / 32	98 / 88	8 / 7	29 / 23	41 / 34	28 / 31	6 / 6
**HR (95%-CI) [e]**	2.55 (1.92-3.39)	8.46 (2.65-26.98)	2.30 (1.34-3.97)	3.10 (2.02-4.74)	2.07 (1.18-3.61)	3.67 (2.75-4.90)	4.02 (1.46-11.08)	4.25 (2.46-7.35)	4.03 (2.56-6.35)	2.95 (1.77-4.92)	3.26 (1.05-10.10)
**HR (95%-CI) [f]**	2.48 (1.86-3.32)	9.07 (2.80-29.45)	2.22 (1.28-3.86)	2.96 (1.92-4.57)	2.11 (1.19-3.73)	3.55 (2.65-4.76)	3.64 (1.30-10.19)	3.92 (2.25-6.84)	3.94 (2.48-6.25)	2.95 (1.75-4.97)	3.34 (1.05-10.58)
**Men**											
**(sub)cohort size, n / N**	2856 / 9786	2856 / 9786	2856 / 9786	2712 / 9153	2469 / 8262	2856 / 9786	2856 / 9786	2856 / 9786	2726 / 9160	2501 / 8283	2856 / 9786
**cases, n / N**	99 / 103	15 / 3	29 / 22	47 / 45	23 / 36	62 / 58	8 / 3	16 / 14	24 / 22	22 / 22	6 / 14
**HR (95%-CI) [e]**	3.13 (2.38-4.13)	17.25 (4.99-59.59)	4.43 (2.55-7.71)	3.40 (2.26-5.13)	2.00 (1.19-3.38)	3.42 (2.39-4.89)	9.27 (2.46-34.96)	3.81 (1.86-7.81)	3.49 (1.96-6.23)	3.10 (1.71-5.60)	1.32 (0.51-3.42)
**HR (95%-CI) [f]**	2.95 (2.22-3.92)	13.43 (3.85-46.89)	3.95 (2.24-6.97)	3.22 (2.12-4.90)	1.97 (1.15-3.36)	3.20 (2.22-4.62)	9.51 (2.46-36.72)	3.94 (1.89-8.23)	3.05 (1.69-5.50)	2.93 (1.60-5.37)	1.18 (0.45-3.10)

Adjusted for: [a] baseline age (only >10–20 years and 20+ years) and sex; [b] baseline age (only >10–20 years and 20+ years); [c] baseline age and sex; [d] baseline age, sex, occupational status, and BMI; [e] baseline age; [f] baseline age, occupational status, and BMI

Table S5: Breslow thickness and Clark´s level at diagnosis of invasive melanoma throughout the study period 1989-2019 in VHM-Skin vs. VHM-Health Exam, vs. *matched* VHM-Health Exam, and vs. the GP-CC by gender. The analyses included BMI and occupational status as additional covariates. IQR, interquartile range; SD, standard deviation

	VHM-Skin		VHM-Health Exam		*P**	*P***	*matched* VHM-Health Exam		*P**	*P***	GP-CC		*P****
**Breslow thickness (mm)**													
	**n**	**median (IQR)**	**n**	**median (IQR)**			**n**	**median (IQR)**			**n**	**median (IQR)**	
**Full cohort**													
**all**	185	0.5 (0.3-0.8)	652	0.6 (0.4-1.3)	<0.05	-	180	0.6 (0.4-1.1)	0.10	-	1443	0.6 (0.4-1.4)	<0.01
**women**	84	0.5 (0.3-0.8)	298	0.6 (0.4-1.2)	0.29	-	91	0.5 (0.3-0.9)	0.91	-	631	0.6 (0.4-1.4)	0.11
**men**	101	0.5 (0.3-0.8)	354	0.6 (0.4-1.3)	<0.05	-	89	0.7 (0.4-1.4)	<0.05	-	812	0.7 (0.4-1.5)	<0.05
**Sub-cohort with additional covariates**													
**all**	165	0.5 (0.3-0.8)	635	0.6 (0.4-1.3)	<0.05	0.09	174	0.6 (0.4-1.0)	0.14	0.16			
**women**	75	0.5 (0.3-0.7)	291	0.6 (0.4-1.2)	0.34	0.44	88	0.5 (0.3-0.9)	0.99	0.99			
**men**	90	0.5 (0.3-0.8)	344	0.6 (0.4-1.3)	0.05	0.12	86	0.6 (0.4-1.4)	<0.05	0.05			
**Clark´s level (1-5)**													
	**n**	**mean ± SD**	**n**	**mean ± SD**			**n**	**mean ± SD**			**n**	**mean ± SD**	
**Full cohort**													
**all**	176	3.0 ± 0.7	630	3.1 ± 0.8	<0.05	-	185	3.2 ± 0.9	0.06	-	1368	3.2 ± 0.9	<0.01
**women**	81	3.0 ± 0.8	295	3.1 ± 0.8	0.36	-	96	3.0 ± 0.9	0.06	-	744	3.1 ± 0.9	0.11
**men**	95	3.0 ± 0.6	335	3.1 ± 0.9	<0.05	-	89	3.3 ± 0.8	<0.01	-	624	3.2 ± 0.8	<0.01
**Sub-cohort with additional covariates**													
**all**	158	3.0 ± 0.7	614	3.1 ± 0.8	0.08	0.17	179	3.2 ± 0.9	<0.05	<0.05			
**women**	72	2.9 ± 0.8	289	3.1 ± 0.8	0.42	0.49	94	3.0 ± 0.9	0.15	0.14			
**men**	86	3.0 ± 0.6	325	3.2 ± 0.9	<0.05	<0.05	85	3.3 ± 0.8	<0.01	<0.05			

* adjusted for baseline age, and sex (all)

** adjusted for baseline age, sex (all), occupational status, and BMI

*** adjusted for baseline age, and sex (all); in the general population, baseline age was age on July 1st, 1993 (see Methods section)

Table S6: Breslow thickness, Clark´s level, and T score at diagnosis of invasive melanoma in VHM-Skin (1989-1994) vs. VHM-Health Exam (1989-2002), vs. *matched* VHM-Health Exam (1989-2002), and vs. the GP-CC (1993-2002) by gender. The analyses included BMI and occupational status as additional covariates. IQR, interquartile range; SD, standard deviation

	VHM-Skin		VHM-Health Exam		*P**	*P***	*matched* VHM-Health Exam		*P**	*P***	GP-CC		*P****
**Breslow thickness (mm)**													
	**n**	**median (IQR)**	**n**	**median (IQR)**			**n**	**median (IQR)**			**n**	**median (IQR)**	
**Full cohort**													
**all**	22	0.7 (0.4-1.4)	93	0.7 (0.4-1.3)	0.71	-	26	0.5 (0.3-0.8)	0.15	-	202	0.7 (0.4-1.3)	0.67
**women**	10	0.7 (0.4-1.2)	49	0.6 (0.4-1.0)	0.65	-	14	0.5 (0.3-0.6)	0.11	-	99	0.6 (0.4-1.0)	0.84
**men**	12	0.7 (0.4-1.5)	44	0.7 (0.4-1.9)	0.92	-	12	0.6 (0.4-0.9)	0.59	-	103	0.8 (0.4-1.6)	0.84
**Sub-cohort with additional covariates**													
**all**	18	0.7 (0.4-1.2)	90	0.7 (0.4-1.3)	0.72	0.74	24	0.5 (0.3-0.8)	0.13	0.13			
**women**	7	0.7 (0.4-0.8)	48	0.7 (0.4-1.1)	0.38	0.36	13	0.4 (0.3-0.6)	0.22	0.22			
**men**	11	0.8 (0.5-1.5)	42	0.7 (0.4-2.0)	0.85	0.67	11	0.6 (0.4-0.8)	0.23	0.31			
**Clark´s level (1-5)**													
	**n**	**mean ± SD**	**n**	**mean ± SD**			**n**	**mean ± SD**			**n**	**mean ± SD**	
**Full cohort**													
**all**	25	3.1 ± 0.8	143	3.0 ± 0.9	0.48	-	42	2.8 ± 0.8	0.24	-	302	3.0 ± 0.9	0.53
**women**	12	3.1 ± 0.8	75	2.9 ± 0.9	0.98	-	25	2.6 ± 0.8	0.34	-	156	2.9 ± 0.8	0.73
**men**	13	3.2 ± 0.8	68	3.0 ± 0.9	0.71	-	17	3.2 ± 0.7	0.98	-	146	3.1 ± 0.9	0.77
**Sub-cohort with additional covariates**													
**all**	21	3.0 ± 0.8	138	3.0 ± 0.9	0.74	0.78	39	2.8 ± 0.8	0.36	0.43			
**women**	8	2.9 ± 0.8	73	2.9 ± 0.9	0.73	0.96	24	2.6 ± 0.8	0.52	0.35			
**men**	13	3.2 ± 0.8	65	3.0 ± 0.9	0.73	0.46	15	3.1 ± 0.7	0.71	0.50			

Table S6: continued

	VHM-Skin		VHM-Health Exam		*P**	*P***	*matched* VHM-Health Exam		*P**	*P***	GP-CC		*P****
**T stage (1-4)**													
	**n**	**mean ± SD**	**n**	**mean ± SD**			**n**	**mean ± SD**			**n**	**mean ± SD**	
**Full cohort**													
**all**	28	2.2 ± 0.8	174	2.1 ± 1.0	0.80	-	46	1.9 ± 0.8	0.14	-	359	2.2 ± 1.0	0.98
**women**	14	2.1 ± 0.9	92	2.1 ± 0.9	0.84	-	26	1.7 ± 0.7	0.31	-	184	2.2 ± 0.9	0.65
**men**	14	2.2 ± 0.8	82	2.2 ± 1.0	0.96	-	20	2.1 ± 0.8	0.76	-	175	2.2 ± 1.0	0.88
**Sub-cohort with additional covariates**													
**all**	24	2.1 ± 0.9	165	2.1 ± 1.0	0.88	0.82	43	1.8 ± 0.8	0.19	0.21			
**women**	10	2.0 ± 0.9	86	2.0 ± 0.9	0.91	0.99	25	1.7 ± 0.7	0.47	0.47			
**men**	14	2.2 ± 0.8	79	2.2 ± 1.0	0.98	0.52	18	2.0 ± 0.8	0.50	0.36			

* adjusted for baseline age, and sex (all)
** adjusted for baseline age, sex (all), occupational status, and BMI
*** adjusted for baseline age, and sex (all); in the general population, baseline age was age on July 1st, 1993 (see Methods section)

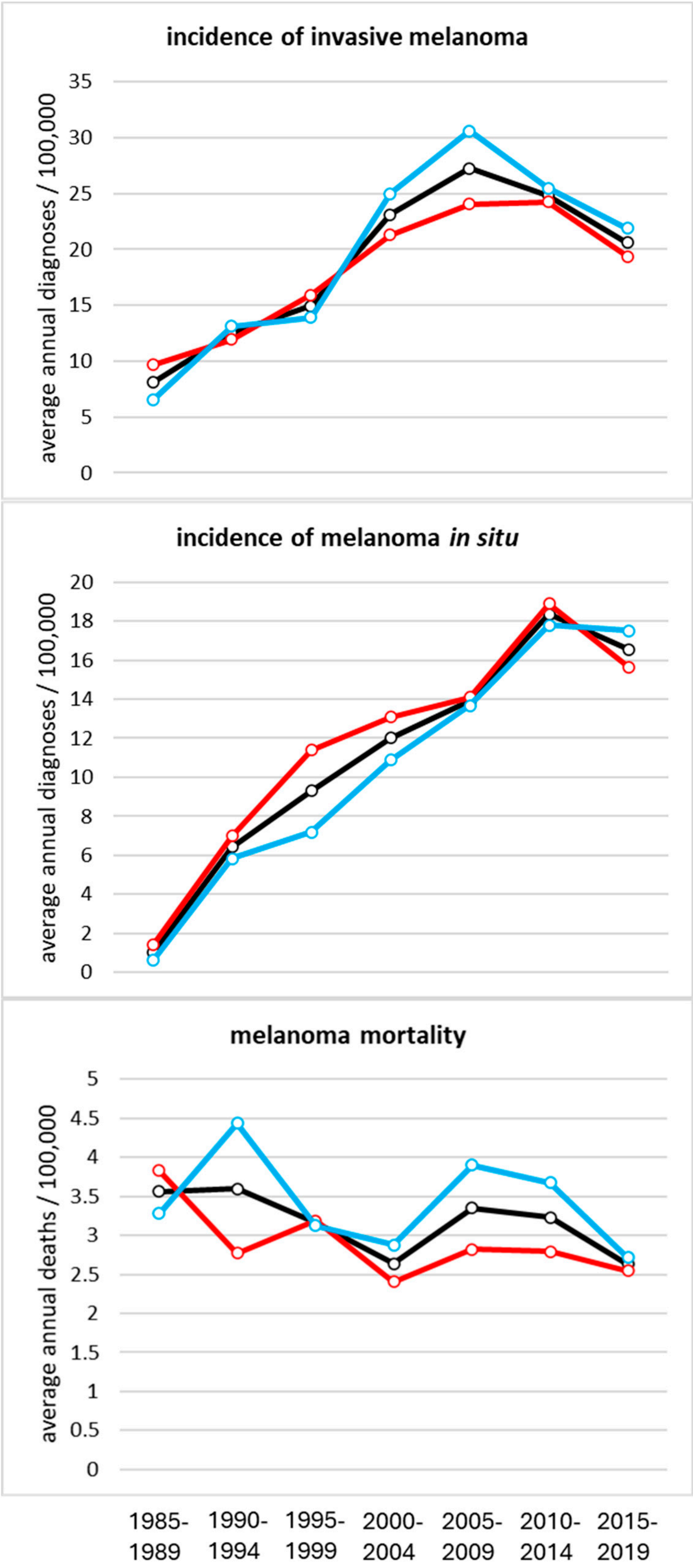

Figure S1: Line graphs showing trends of age-adjusted invasive melanoma incidence, melanoma *in situ* incidence, and melanoma mortality in the general population of Vorarlberg in 5-year intervals 1989-2019. Line colors: black, all; red, women; blue, men
